# University of Patanjali as a Centre of Excellence in Yoga Education

**DOI:** 10.1016/j.jaim.2026.101404

**Published:** 2026-10-13

**Authors:** Rudra B. Bhandari, Sadhvi Devpriya

**Affiliations:** a School of Yoga Sciences, Swami Rama Himalayan University, Dehradun, Uttarakhand, 248016, India; b Faculty of Humanities and Ancient Studies, University of Patanjali, Haridwar, Uttarakhand, India

**Keywords:** Yoga education, Learning excellence, Wellness, Personality development, Social empathy

## 1 Introduction

The University of Patanjali (UOP) envisioned and established by Yoga Master Swami Ramdev and Ayurveda Scholar Acharya Balkrishna, named after the sage Patanjali (c. 900 BC), established through Act No. 4/2006 of the Uttarakhand State Legislature, published in the State Gazette on April 05, 2006, and funded by Patanjali Yogpeeth (PYP), is a Yoga University that aims to carry out quality teaching, learning, research, and advocacy on Yoga, Ayurveda, Vedic wisdom, and culture to produce professionally competent, personally developed, and socially empathetic youths dedicated to promoting Indian Knowledge System (IKS). UOP engaged the community people, educationists, traditional knowledge workers, philosophers, theologians, sociologists, philanthropists, psychologists, scientists, administrators, politicians, bureaucrats, policy makers, health workers, technocrats, and corporate houses as its stakeholders to explore educational gaps/needs, identify potential learners, design and implement curricula and course contents, guide teaching-learning process and activities, and evaluation schemes.

The UOP approved under Section 2f of the UGC Act 1956 and Section 35 (1) (iii) of the Income Tax Act has been recently accredited by the National Assessment and Accreditation Council (NAAC) on September 26, 2024, with a grade A^+^ and a cumulative grade point average (CGPA) of 3.48 in its first cycle evaluation as a Centre of Excellence in Yoga Education (COEYE). UOP's vision is to integrate *Vedic* wisdom and scientific research to provide operational solutions to contemporary global challenges and to produce creative, skilled, healthy, prosocial, and globally well-versed human resources through *Vedic* living, attitude, attributes, global citizenship, and leadership behaviors. To translate vision into action and outcomes, UOP has designed and implemented higher education on Yoga, Ayurveda, *Vedic* knowledge, and culture, along with modern scientific and technological inputs, to validate, preserve, and promote them. The establishment of conducive infrastructure and a psychosocial ambience for the implementation and inculcation of *Vedic*, yogic, and spiritual principles and practices among institutional stakeholders may enable them to contribute to building inclusive, peaceful, and sustainable societies aligned with the United Nations (UN) Sustainable Development Goal (SDG) 4.7 also comes under UOP's mission.

The UGC inspection was held from March 12 to March 14, 2021, and the Choice-Based Credit System (CBCS) was implemented from the 2020-21 academic session. UOP has implemented the New Education Policy 2020 (NEP 2020) at the undergraduate level in the academic session 2022-23. NEP 2020 aligns with global trends focused on competency-based education and experiential learning, aiming to improve learners' outcomes, empower teachers, and strengthen educational infrastructure, thereby promoting equity, quality, and innovation to overcome the existing challenges of the Indian education system [1].

This study covers UOP's 5-year longitudinal data account from June 01, 2017, to December 31, 2022, to project NAAC's seven quality indicators related to the Yoga University: 1) Curricular aspects, 2) Teaching-learning and Evaluation, 3) Research, innovations, and extensions, 4) Infrastructure and learning resources, 5) Student support and progression, 6) Governance, leadership and management; and 7) Institutional values and best practices to highlight it as the COEYE. Thus, this study aims to depict and discuss the UOP as a COEYE, based on summarized data captured from academic sessions 2017-18 to 2021-22 regarding seven quality indicators of the Yoga University, as set by NAAC.

## 2 Contributions in yoga education and research

The UOP exemplifies a strategic convergence of IKS with formal higher education, as evidenced by its A+ grade in the NAAC's first evaluation. Across seven NAAC criteria, the university has progressively aligned its curriculum, pedagogy, research, leadership, governance, infrastructure, and best practices with national imperatives of the NEP 2020. This section provides an analytical discussion of the institution's performance on each criterion, with a specific emphasis on the Department of Yoga Science's (DYS's) highest contributions to UOP's academic and research base.

### 2.1 Curricular aspects

Between 2017 and 2022, UOP expanded its programs from 18 to 31, guided by outcome-based curriculum reforms that drew on both Vedic knowledge systems and global competency standards. The DYS led this transformation by offering seven uniquely structured programs that integrated program specific outcomes (PSOs) and course outcomes (COs), focusing on experiential learning, Sanskrit texts, and traditional therapy, thereby demonstrating alignment with the NEP's call for multidisciplinary and holistic education [2]. This experiential integration addresses global calls for re-indigenizing knowledge systems in higher education [[3], [4], [5]]. The Yoga lifestyle practices and academic activities (scheduled from 04 a.m. to 10 p.m.), daily *yajna*, vegetarian dietary habits, subsequent guest deliberations of notable and quotable figures, lucrative financial incentives for memorization of IKS-related classical texts, subsequent national and international programs and deliberations from its founder leaders targeting learners are found effective for their professional and personal transformation.

Compared to quotable Yoga institutions such as Swami Vivekananda Yoga Anusandhana Samsthana(SVYASA) (Bangalore), which follows an integrated approach to Yoga therapy (IAYT) model blending biomedical and health sciences with Yoga interventions [6], UOP emphasizes classical textual depth through Sanskrit exegesis and classical commentary. Similarly, while the Morarji Desai National Institute of Yoga (MDNIY) operates under the Ministry of AYUSH, focusing on large-scale training and certification programs [7], UOP differentiates itself by prioritizing philosophical rigor and therapeutic research grounded in the IKS.

Thus, while other institutions specialize in clinical therapy (SVYASA) and large-scale training (MDNIY) [7], UOP offers a comprehensive model that combines well-defined pedagogy, academic rigor, value-based instruction, research, and Vedic lifestyle integration, making it a distinctive institution within the Indian higher education ecosystem. This model aligns with the United Nations Educational, Scientific, and Cultural Organization (UNESCO)'s global call for a “new social contract for education,” which places well-being, ethics, and collective learning as the focal point of curriculum renewal [8,9].

### 2.2 Teaching–learning and evaluation

The UOP adopted an integrative teaching and learning ecosystem that combines experiential pedagogy, vocational courses, value-based mentorship, and structured evaluation. The institution followed a 70:30 ratio of summative to formative assessment, in line with UGC's quality mandate for competency-based education [10].

The DYS distinguished itself through a pedagogy that fused classical instruction, textual exegesis, and therapeutic exposures. Students participate in scriptural debate, guided chanting, daily Yoga lifestyle practices, and field-based assignments. This integration of textual depth with ethical practice supports NEP's vision for holistic growth [11]. Recently published, the Yogic Personal Excellence Inventory (YPEI), developed and validated by Bhandari and Chaudhry [12] at the DYS, is a notable contribution to Yoga psychology aligned with UOP's pedagogical ethos.

A comparative view highlights that institutions like SVYASA and MDNIY emphasize the clinical application of Yoga with strong ties to allied medical sciences and AYUSH institutions. However, these models tend to emphasize biomedical validation and physical techniques over value-based pedagogy or philosophical literacy. Dev Sanskriti Vishwavidyalaya (DSVV) employs a holistic teaching model that is rooted in value education, cooperative learning, and community service [13]. DSVV's emphasis on moral and civic education aligns with the NEP's graduate attribute framework; however, its content delivery focuses more on character development than on textual scholarship or therapeutic training. UOP's pedagogical structure strikes a balance by integrating yajna, Yoga therapy, lifestyle management, Sanskrit grammar, and research-oriented assignments.

While the university maintained an average final-year pass rate of 88.6%, the DYS consistently recorded pass rates above 92%, and 38% of students achieved distinctions in the 2021–22 academic year alone. The Yoga curriculum helped 52 alumni qualify for the NET/JRF exam over five years, a prerequisite for advanced research and academic careers.

### 2.3 Research, innovation, and extension

UOP demonstrated a growing institutional focus on scholarly output, traditional knowledge preservation, and community-oriented extension during its first NAAC cycle. Between 2017 and 2022, the university produced 535 research papers, with an i-index of 479 and an h-index of 56, and secured 16 patents, primarily in the domains of Ayurveda, Yoga therapy, Sanskrit linguistics, and other traditional medicines. Research activities were aided by the Research Development Cell (RDC) and reviewed/monitored by the institutional ethics committee (IEC) and the internal quality assurance cell (IQAC). The university is committed to exploring, describing, analyzing, validating, and innovating the IKS.

The DYS contributed 223 research publications, accounting for over 40% of the university's total output. These included empirical studies in Yoga therapy, psychophysiology, philosophical analyses of Yogic texts, and documentation of indigenous lexicons. The DYS also produced 209 postgraduate dissertations and 94 creative writing projects, indicating its key role in the university's research landscape. Compared to other Yoga universities such as SVYASA, which emphasizes interventional studies and psychometric testing in collaboration with institutions like the National Institute of Mental Health and Neurosciences (NIMHANS) and the Indian Institute of Science (IISc), UOP maintains a distinct *Shastric-*philosophical orientation. Its contributions include the *Saumitreya* corpus, Ayurvedic-Yogic plant dictionaries, and documentation of 6.3 million digitized manuscript pages. These initiatives contribute to what scholars describe as “epistemic sovereignty”—the preservation and systematization of non-Western knowledge forms [14].

UOP's extension activities are grounded in the principle of public well-being and welfare. The DYS led more than 40 outreach initiatives, including school-based Yoga literacy, prisoners' wellness, and elderly health, aligning closely with SDG 3 (Health and Well-being) and SDG 4 (Inclusive Education) [9,15].

While UOP has collaborated with Patanjali Research Foundation Trust and Patanjali Wellness Organizations to cater clinical exposure and research data generation facilities for the students, in-house set up of polyclinic with well equipped labs may motivate and aid students for practical learning and the conduct of the research.

### 2.4 Infrastructure and learning resources

During the assessment period, UOP enhanced its physical and digital infrastructure to support teaching and research. By 2022, over 60 classrooms were operational, with 82% ICT-enabled. The central library housed more than 11,000 books and provided access to Shodhganga, NDLI, and INFLIBNET. These developments align with the UGC's vision for digital transformation and NAAC's expectations under Criterion 4 for learning resource modernization [16].

The DYS maintained a dedicated block with smart classrooms, a yoga therapy lab, and access to clinical facilities at Patanjali Wellness. It also curated a digitized manuscript archive exceeding 6 million pages, integrating heritage preservation into the learning process. Compared to MDNIY, which offers primarily clinical infrastructure, and DSVV, which focuses on value-based residential facilities, UOP's Yoga infrastructure combines Vedic lifestyle, academic, research, and technological components [17].

### 2.5 Student support and progression

From 2017 to 2022, the UOP disbursed over INR 24.69 lakhs in scholarships to 218 students through various schemes, including the Laxmi Charitable Trust, covering both merit-based and need-based criteria.

The DYS recorded 198 placements across AYUSH centres, wellness clinics, and research institutions and produced 52 NET/JRF qualifiers, demonstrating strong PG teaching-learning. Students received national recognition in events such as Khelo India and the National Yogasana Championship. This model of structured academic support resonates with global higher education models that prioritize progression through advisory scaffolding and academic coaching [18]. These outcomes reflect NEP's goals of ensuring holistic development, social equity, and academic progression [2] and align with studies emphasizing the impact of targeted financial and academic support in improving student outcomes [19,20].

### 2.6 Governance, leadership, and management

The UOP has implemented a structured governance system, supported by e-governance platforms, in planning, admissions, finance, and evaluation. An active IQAC monitored academic standards and coordinated quality improvement initiatives. SOPs were developed for curriculum development, grievance redressal, and performance appraisal. The DYS applied additional SOPs for therapy protocols and mentorship. Faculty appraisal included teaching outcomes, research performance, and community services.

Student representation in the IQAC and academic sub-bodies ensured participatory decision-making, aligning with the UGC's quality mandate for inclusive governance [21]. The leadership philosophy at UOP resonates with the Yogic Leadership model developed by Sangodkar and Bhandari [22] which includes multiple intelligences —bodily, cognitive, emotional, and spiritual —and wellness dimensions aligned with UOP's ethos. These practices align with global models of transformational leadership in higher education, where collaboration, well-being, and accountability are key to institutional resilience [23]. However, undefined recognition, regulatory rules and regulations on higher Yoga education, weak motivations for Yoga learning, an uncertain market for Yoga professionals, limited start-up institutional funding, and poor infrastructure were the initial challenges faced by UOP. The increasing mobilization of UOP alumni into self-employment/job placements motivated learners to pursue higher Yoga education. The initial infrastructural constraints were resolved when UOP began teaching and learning at its new campus, inaugurated by Hon'ble Amit Shah, Home Minister of India, on March 31, 2023.

Implementation of well defined, objective, rigorous, transparent and engaging SOPs regarding 1) teaching-learning mechanisms and practices for boosting learners' creativity and innovation and their learning outcomes' evaluation; 2) teachers' identification, evaluation, recruitment, pay structure, continuous appraisal and promotion strategies; 3) critical, rigorous and anonymous review of research proposals, UG Dissertations, and PhD theses to ensure/promote quality and proportionate publications across all existing departments may add value to translate UOP's foundational plan and vision into actions and outcomes.

### 2.7 Institutional values and best practices

UOP's culture is grounded in Vedic wisdom, Yoga lifestyle, inclusive management, and Indian spirit. Its guiding philosophy is shaped by the Yogic vision of holistic development, national well-being, and cultural preservation—all of which correspond to NAAC Criterion 7 and the transformative ethos of NEP 2020. The university promotes inclusivity through its core tenet of the world as a family, irrespective of caste, creed, gender, socio-economic status, or region. This is reflected in equitable scholarship policies, gender-balanced enrolment, and participatory student governance.

Daily Yoga practices and *yajna*, incentivized scriptural memorization, and lifestyle management education are embedded in all students' lives. UOP's model of integrating these practices into daily academic life, as conceptualized by Chaudhry et al. [24], facilitates the yogic perspective on personal excellence. Environmental practices include rainwater harvesting, waste segregation, and a plastic-free academic zone, which fulfil NAAC's sustainability benchmarks and contribute to SDGs 6, 12, and 13 [15].

Students gain exposure to digital communications, public speaking, and social media literacy through several events covered and telecasted on Aastha, Sanskar, and Vedic Broadcasting channels. These practices align with the UNESCO framework for socially engaged and culturally grounded higher education [8,25], positioning UOP as a model for an integrative value-based institution.

### 2.8 Limitations, future plans directions

UOP has made substantial progress in curricular design, value-integrated pedagogy, and Yoga-centered research. However, some structural and operational limitations must be acknowledged and addressed to realize its full potential as a COEYE and the IKS.

Although the DYS is committed to developing well-equipped therapy labs and providing access to clinical facilities, the entire university has yet to operationalize in-house laboratories and interdisciplinary patient care units. These are essential not only for enhancing practical learning but also for promoting empirical, and evidence-based research across all departments.

Secondly, the institutional framework for employee well-being, professional growth, and performance appraisal needs improvement. There is a need for transparent evaluation benchmarks, structured academic audits, and credible mechanisms for feedback and accountability. Ensuring a consistent culture of academic leadership development through incentives, appraisals, and targeted capacity-building will be critical to maintaining long-term human resource empowerment, enhancing organizational commitment, and reducing attrition.

Another area requiring strategic focus is the uneven implementation of SOPs in academic, administrative, and research activities. Although the IQAC has introduced guidelines in certain areas, a comprehensive SOPs covering academic planning, admissions, assessment, grievance redressal, and research ethics still needs substantial improvement. Institutionalizing SOPs will reinforce consistency, compliance, and quality work culture across all units/wings/departments needed for sustainable success.

In terms of curriculum, while the Faculty of Yoga has achieved a commendable integration of traditional wisdom with contemporary knowledge and skills, there is a pressing need to revise older curricula across other departments targeting critical thinking and problem solving. The goal must be to ensure all programs are technologically relevant, career-focused, and infused with ethical, social, and ecological consciousness. Promoting digital fluency, professional competency, global awareness, and cross-disciplinary learning will position learners as adaptable professionals with a more profound sense of social empathy and responsibility, aligned with the university's vision.

To translate the university's vision of creating professionally competent, personally developed, socially empathetic, and nationally committed human resources into action, a university-wide outreach strategy is required to integrate community engagement and services, holistic and multidisciplinary teaching-learning, research and innovation, and partnerships with national and international institutions.

UOP's aspiration to produce contributors to multidisciplinary education, entrepreneurship, healthcare, and life management requires a sustained and strategic focus on expanding applied research, improving academic leadership, strengthening digital and internationalization strategies, and embedding institutional mechanisms that support continuous quality improvement.

## 3 Conclusions

This article highlights UOP's developmental trajectory during its first NAAC accreditation cycle, offering a five-year longitudinal account of curricular, teaching-learning, and evaluation, research and development, infrastructure and learning resources, student support and empowerment system, management, values, and best practices in yoga-based higher education. The analyses of seven core criteria reveal that the university has established a robust foundational model grounded in IKS, prioritizing Yoga Science. The synthesis of traditional values with structured academic frameworks has enabled the university to position itself as a distinctive institution in the landscape of Yoga and allied IKS-related teaching and learning. This study projects UOP's potential to make meaningful contributions to national priorities in holistic health, value-based learning, educational transformation, and the revival of the IKS. This may be an imperative case for other universities that aspire to excellence in an IKS-grounded education system. However, critical observations on UOP's strengths, achievements, and limitations highlight the need for continuous improvement across strategic plans, processes, and practices, as well as curricula development, technological innovations, professionalism, employee well-being, teaching-learning and evaluation mechanisms, placement schemes, and community engagement activities. UOP's foundational plan and vision to integrate IKSs with modern science and technology via higher teaching-learning and quotable interdisciplinary scientific research warrants equal focus on teaching-learning and research on basic, applied, social, medical, and premedical sciences; engineering and technology for the scientific validation of principles and practices of Yoga, Ayurveda, and other IKS spectrums for their bio-psycho-socio-spiritual implications to overcome personal, professional, societal, ecological, and spiritual problems encountered at contemporary times.

## Author contribution statement

**R.B·B:** Conceptualization, Methodology/Study Design, Investigation, Data Curation, Visualization, Validation, Software, Resources, Formal Analysis, Writing-original draft, Writing-review and editing, Funding acquisition.

**S.D:** Validation, Supervision, Project Administration.

## Use of AI declaration

The authors declare that no AI was used in the manuscript preparation.

## Funding sources

The authors did not receive any funding to carry out the study.

## Conflict of Interest

The authors declare the following financial interests/personal relationships which may be considered as potential competing interests:Rudra B. Bhandari reports financial support and administrative support were provided by University of Patanjali. Rudra B. Bhandari reports a relationship with Swami Rama Himalayan University that includes: employment.
