# Supplementary material for: University of Patanjali as a Centre of Excellence in Yoga Education
Source: J Ayurveda Integr Med. 2026 Oct 13;17(5):101404. doi: 10.1016/j.jaim.2026.101404 (PMC13681613; doi:10.1016/j.jaim.2026.101404)
Supplement: Supplementary file 1 [file mmc1.docx]

**2. Method**

***2.1 Study Design***

This study was designed, conducted, recorded, and reported longitudinally, covering academic session-wise data from June 01, 2017, to December 31, 2022, on seven quality indicators of Yoga Education. To collect the data, we employed a range of methods, including overt and covert observations, group discussions, interviews, seminars, conferences, semester-end and formative evaluation outcomes, laboratory visits, fieldwork, projects, internships, co-curricular and extracurricular activities, lifestyle assessments, and feedback from students, guardians, and employees. The Institutional Ethics Committee (IEC) and the Internal Quality Assurance Cell (IQAC) of the University of Patanjali approved this study.

***2.2 Study Participants***

Study participants included students, teaching and non-teaching staff, parents, external board and committee members, conference, seminar, symposium, and workshop speakers, and other stakeholders.

***2.3 Sources of Data Collection***

To collect the data we accessed the minutes of the board of studies, the academic council, the board of management, board of faculty, finance committee and the board of governors, curricula and course contents, All India Survey of Higher Education (AISHE) reports, IQAC records, operational teaching-learning activities, alumni and guardians’ and teachers’ feedback, rules and guidelines of NEP 2020, admission records, evaluation records, field work, internship, hospital, hostel, financial, research publication, community outreach, NSS, NCC and placement records, records of IEC, research cell, disciplinary committee, grievance redressal cell, anti-ragging committee, mentor-mentee practice, awards/rewards/recognition, guest deliberations, information and communication technology (ICT) and sports facilities, library, faculty development programs and national/international activities, memorandum of understandings (MoUs) signed between UOP and other organizations, human resources records, co-curricular and extracurricular records, lifestyle pattern of the organizational staff, fire safety and waste management process and practices, media exposures, print and electronic medias, festive and patriotic celebrations.

***2.4 Data Analyses***

We computed descriptive statistics (counts, frequencies, mean, and standard deviation) to depict and compare the results of the data regarding five consecutive academic years from 2017-18 to 2021-22, using graphs, bar diagrams, and regional clusters.

**3. Results**

***3.1 Curricular Aspects (2017–2022)***

The University of Patanjali expanded its academic offerings from 18 programs in 2017–18 to 31 programs in 2021–22, accompanied by an increase in courses from 315 to 421 during the same period (Figure 1). This academic expansion was supported by a shift to Outcome-Based Education (OBE) and the Choice-Based Credit System (CBCS). Curriculum outcomes were aligned with Program-Specific Outcomes (PSOs) and Course Outcomes (COs), structured around a Lecture–Tutorial–Practical (L:T:P) mode of delivery.

**Figure 1. Growth in Number of Programs and Courses from 2017 to 2022.**

|  | No. of Programs | No. of Courses |
| --- | --- | --- |
| 2017–18 | 18 | 315 |
| 2018–19 | 20 | 377 |
| 2019–20 | 21 | 407 |
| 2020–21 | 25 | 391 |
| 2021–22 | 31 | 421 |

As of 2022, UOP comprises four faculties and ten departments (Table 1). The Department of Yoga Science (DYS), under the Faculty of Yoga and Health, was established in 2009 and currently offers seven academic programs with a total of 627 enrolled students and 27 staff members (Table 2). DYS’s yearly average intake including certificate, post graduate diploma, undergraduate, post graduate and PhD programs was 346 over five academic years covered in NAAC assessment period (2017 to 2022), i.e. 326 for each consecutive academic year 2017-143 18, 2018-19, and 2019-20 and 375 for each consecutive academic year (2020-21 and 2021-22).

Faculty strength has expanded over time, with notable growth in both academic and instructional positions (Table 3).

**Table 1: List of Faculties and Associated Departments (as of 2022)**

| **Faculty** | **Departments** |
| --- | --- |
| Yoga and Health | 1. Department of Yoga Science 2. Department Physical Education and Sports |
| Humanities & Ancient Studies | 3. Sanskrit  4. Philosophy  5. Psychology  6. Travel & Tourism Management  7. Music  8. English |
| Science | 9. Allied & Applied Science |
| Naturopathy & Yogic Sciences | 10. Naturopathy & Yogic Sciences |

**Table 2: Department of Yoga Science – Faculty and Program Profile (as of 2022)**

| **Attribute** | **Details** |
| --- | --- |
| Faculty Name | Faculty of Yoga Science |
| Department | Department of Yoga Science |
| Year of Establishment | 2009 |
| Total Staff | 27 |
| Total Students Enrolled | 627 |
| Academic Programs Offered | 7 |

**Table 3: Faculty Composition**

| **Designation** | **Assessment Year** | **Current Year (2022)** | |
| --- | --- | --- | --- |
|  | **DYS** | **UOP** | **DYS** |
| Professors | 02 | 09 | 04 |
| Associate Professors | 00 | 02 | 01 |
| Assistant Professors | 08 | 37 | 07 |
| Yoga Instructors | 07 | 0 | 15 |
| Total Faculty | **17** | **48** | **27** |

Between 2017 and 2022, a total of 49 faculty members across the university participated in curriculum design, including 16 from the DYS. Of the four institution-wide curriculum revision workshops, three were organized by the DYS. The department also introduced seven NEP 2020-aligned courses, nine value-added or skill-based courses, six interdisciplinary courses, and 15 new electives, contributing to seven of the 13 total programs newly launched or revised across the university during this period (Figure 2).

**Figure 2: Comparison of Curricula Updates in the University vs. Department of Yoga Science from 2017 to 2022.**

|  | University-Wide (All Departments) | Yoga Department Only |
| --- | --- | --- |
| Total Programs Introduced/Revised | 13 | 7 |
| New Electives Added | 38 | 15 |
| Interdisciplinary Courses Developed | 12 | 6 |
| Value-Added / Skill-Based Courses Introduced | 25 | 9 |
| Courses Aligned with NEP 2020 Outcomes | 31 | 7 |
| Curriculum Revision Workshops Held | 4 | 3 |
| Faculty Participated in Curriculum Design | 49 | 16 |

***3.2 Teaching–Learning and Evaluation***

UOP employed a student-teacher ratio of 19:1, maintaining a 70:30 balance between external and internal evaluations. Assessment formats included written exams, practical, presentations, and viva voce.

In the 2021–22 academic year, students were enrolled from 17 Indian states (Figure 3), with a demographic split of approximately 60% male and 40% female. In 2021–22, the DYS enrolled over 120 students across its core programs, with a five-year cumulative enrolment of 301 in MSc Yoga, 257 in MA Yoga, 200 in BA Yoga, and 195 in BSc Yoga, indicating consistent growth and program demand over time (Figure 4).

**Figure 3. Geographical Distribution of the Students Enrolled in the Academic Session 2021-22.**

**Figure 4. Student Enrolment in Yoga Programs from 2017 to 2022.**

|  | BA Yoga | BSc Yoga | MA Yoga | MSc Yoga |
| --- | --- | --- | --- | --- |
| 2018–19 | 57 | 62 | 76 | 100 |
| 2019–20 | 60 | 55 | 66 | 72 |
| 2020–21 | 50 | 43 | 72 | 81 |
| 2021–22 | 33 | 35 | 42 | 48 |
| Total | 200 | 195 | 257 | 301 |

The instruction pattern in the DYS included 30% theoretical instruction, 50% practical, 10% chanting, and 10% field assignments. The DYS led the way in digital readiness, with all faculty members completing Ayurveda, Yoga, Unani, and Siddha (AYUSH)-based e-content and Massive Open Online Course (MOOC) certifications.

The institutional final-year pass rate averaged 88.6%, while the DYS consistently reported rates above 92%, with 38% of students receiving distinction in 2021–22 alone (Figure 5). These performance trends indicate effective academic mentoring and learners’ engagement with the curriculum and courses.

**Figure 5. Year-wise Final Year Students’ Performance (2017–2022)**

|  | Appeared | Passed | Pass Percentage (%) |
| --- | --- | --- | --- |
| 2017–18 | 300 | 269 | 89.67 |
| 2018–19 | 430 | 341 | 79.3 |
| 2019–20 | 412 | 366 | 88.83 |
| 2020–21 | 292 | 284 | 97.26 |
| 2021–22 | 307 | 238 | 77.52 |

***3.3 Research, Innovation, and Extension***

Between 2017 and 2022, UOP produced 535 research publications, achieving an i-index of 479 and an h-index of 56 (Figure 6). These outputs were coordinated through the Research Development Cell (RDC) and IQAC’s quality assurance mechanisms.

**Figure 6. Year-wise Trend in Research Publications from 2017 to 2022.**

|  | Research Papers | i-index | h-index |
| --- | --- | --- | --- |
| 2017–18 | 61 | 54 | 7 |
| 2018–19 | 51 | 44 | 7 |
| 2019–20 | 42 | 28 | 14 |
| 2020–21 | 105 | 91 | 14 |
| 2021–22 | 276 | 262 | 14 |
| Total | 535 | 479 | 56 |

The DYS led institutional research, resulting in 223 publications, including original studies in Yoga and Ayurvedic lexicons. Additionally, it produced 209 Master’s dissertations (i.e., 63, 33, 35, 48, and 30 in respective academic sessions from 2017-18 to 2021-22) supported 94 creative writing submissions and filed 16 patents, and enabled 52 students to qualify for National Eligibility Test (NET)/Junior Research Fellowship (JRF) exams (10 NET and 42 JRF) (Figure 7).

**Figure 7. Research Achievements of the Department of Yoga Science from 2017 to 2022.**

|  | Research Projects | Research Publications | Patents | Dissertations | Book with ISBN | Creative Writing | PhD Degree Awarded | NET+JRF |
| --- | --- | --- | --- | --- | --- | --- | --- | --- |
| 1 | 6 | 223 | 16 | 209 | 101 | 94 | 14 | 52 |

Significant scholarly contributions included the digitization of 6.3 million manuscript pages, the development of Ayurvedic plant dictionaries, and the composition of 200,000 Sanskrit verses. The department’s Saumitreya corpus, which documents over 21,000 medicinal plants, represents one of India’s most significant lexicographic projects in the integration of Yoga and Ayurveda. More than 40 community outreach programs were conducted, including school Yoga and literacy, women’s health, prisoners’ wellness, and community nutrition awareness activities. As of 2022, the DYS research publication record showed strong individual contributions. Prof. Acharya Balkrishna had the highest publication count, 47, followed by Prof. Shirley Telles with 39 publications. The other 21 publications included nine by Dr. Rudra Bhandari and six by each Dr. Arti Yadav and Dr. Sandeep Kumar Singh (Figure 8).

**Figure 8. Faculty-wise Research Publication Counts in Department of Yoga Science as of 2022.**

|  | Publication Count |
| --- | --- |
| 1 Prof. Acharya Balkrishna | 47 |
| 2 Prof. Om Narayan Tiwari | 0 |
| 3 Prof. Paran Gowda | 0 |
| 4 Prof. Shirley Telles | 39 |
| 5 Dr. Rudra Bhandari | 9 |
| 6 Dr. Anju Kumari | 0 |
| 7 Dr. Arti Yadav | 6 |
| 8 Dr. Sandeep Kumar Singh | 6 |
| 9 Dr. Aarti Pal | 0 |
| 10 Dr. Monika Sharma | 0 |
| 11 Dr. Ankit Kumar | 0 |
| 12 Shree Subhash | 0 |

***3.4 Infrastructure and Learning Resources***

As of 2022, UOP operated over 60 classrooms, with 82% equipped with innovative technology and access to the Information and Library Network Centre (INFLIBNET), Shodhganga, and the National Digital Library of India (NDLI). A library with 11,000+ books supported blended academic delivery. The university adopted e-governance tools across admissions, finance, and exam management.

The DYS had a dedicated facility with smart classrooms, a Yoga Therapy Lab, a Yajna Shala, and digitized manuscript terminals, enabling ritual instruction alongside technology-based learning. Its infrastructure also included simulation spaces for yoga therapy and scriptural interpretation.

Faculty from all departments participated in digital pedagogy workshops, with the Yoga faculty achieving 100% participation in MOOC creation and AYUSH-based e-learning certifications.

***3.5 Student Support and Progression***

Between 2017 and 2022, the UOP sponsored a total of INR 24.69 lakhs merit-based scriptural memorization rewards and need-based scholarships (e.g., Laxmi Charitable Trust) to 218 students.

The DYS demonstrated strong performance in graduate outcomes. Over the five years, 198 students from the DYS were placed in wellness centres, AYUSH institutions, research bodies, and public services.

The DYS also hosted career workshops in collaboration with Patanjali Wellness and Patanjali Research Foundation, offering mentorship in resume writing, field-based therapy, personality development, research capacity-building, and opportunities for postgraduate fellowships. Student achievements extended beyond academics. A total of 63 institutional awards were received by students and faculty, including top honours at events such as the Khelo India University Games and the National Yogasana Championships, as well as a listing in the Golden Book of World Records. The Vice-Chancellor received international recognition for contributions to healthcare and education. During the same period, a consistent number of students qualified in national-level competitive examinations. The highest number of students was qualified in 2018–19, with 532 students, followed by 2019–20 (489), 2017–18 (483), 231 2021–22 (423), and 2020–21 (408) as depicted in Figure 9.

**Figure 9. Students Qualified in Competitive Exams**
